# Technostress and generative AI in the workplace: a qualitative analysis of young professionals

**DOI:** 10.3389/frai.2025.1728881

**Published:** 2025-12-12

**Authors:** Malte Högemann, Laura Hein, Jan-Oliver Britsche, Oliver Thomas

**Affiliations:** 1 Department of Information Management and Business Informatics, University Osnabrueck, Osnabrueck, Germany; 2 Smart Enterprise Engineering, German Research Centre for Artificial Intelligence GmbH (DFKI), Osnabrueck, Germany

**Keywords:** ChatGPT, Generative AI, qualitative study, technostress, young professionals

## Abstract

Generative artificial intelligence (GenAI) is rapidly diffusing into the workplace and is expected to substantially reshape roles, workflows, and skill requirements, particularly for young professionals as early adopters who are highly exposed to these tools. While GenAI is widely regarded as a means to increase productivity, its adoption may simultaneously introduce new challenges, including various forms of technostress. Drawing on 15 semi-structured interviews with young professionals in research and development (R&D), IT, finance, and marketing in organizations piloting or using GenAI, we conducted a structured qualitative content analysis guided by established technostress dimensions. Our findings indicate that classic technostress dimensions remain relevant but manifest differently across sectors and contexts. Moreover, additional GenAI-specific stressors emerged, such as regulatory and compliance ambiguity, data protection and copyright concerns, perceived dependency, potential skill degradation, doubts about the reliability and controllability of AI outputs, and a shift towards more monitoring and conceptual work. At the same time, participants reported techno-eustress in the form of efficiency gains, learning opportunities, and enhanced intrinsic motivation. Overall, the study extends existing technostress frameworks and underscores the importance of AI literacy, clear organizational governance, and supportive work design to mitigate negative technostress while enabling the productive use of GenAI.

## 1 Introduction

The rapid development and adoption of GenAI, particularly ChatGPT, is reshaping many industries, leading to significant changes in skill requirements and job profiles (Gmyrek et al., 2023; Sedkaoui and Benaichouba, 2024). While headlines in the popular media often paint a rather sensational picture of inevitable job losses due to GenAI (Butler, 2025; Mauran, 2023), the actual impact on the labor market is far more complex (Jung and Desikan, 2024; World Economic Forum, 2025). Studies by organizations such as McKinsey, the World Economic Forum, and UK research institutes predict that the sectors most susceptible to automation and AI applications, such as finance, marketing, research & development (R&D) and IT, will experience significant job shifts, with some roles becoming obsolete and new ones emerging (Chui et al., 2023; Ellingrud et al., 2023; Jung and Desikan, 2024; World Economic Forum, 2025). An initial study by Upwork with 2,500 participants shows that 96% of managers expect higher productivity from the use of GenAI, while 47% of workers struggle to meet these higher expectations, and also as many as 77% of subordinates feel that AI tools decrease their productivity or even increase their workload (Monahan and Burlacu, 2024). Other studies have indicated that young employees are particularly inclined to utilize GenAI in their professional settings (Schlude et al., 2024). Beyond recent studies on GenAI use, research on age and technology adoption indicates that younger workers tend to be more open to experimenting with new digital tools. Hauk et al. (2018) found that age was negatively related to perceived ease of use, perceived usefulness, and intention to use new technologies. Morris and Venkatesh (2000) demonstrate that younger employees are more willing to engage with and try out novel technologies in a workplace setting. These findings suggest that younger cohorts are not only highly exposed to GenAI but are also particularly likely to adopt and integrate it into their work. Therefore, it is essential to understand how GenAI affects the stress of this demographic in order to address both individual and organizational challenges. A particular form of stress that has been associated with the adoption of digital technologies, including GenAI, is technostress. This phenomenon, originally conceptualized by Brod (1984) and further developed by Tarafdar et al. (2007), provides a valuable tool for examining the negative effects of technology adoption. While previous qualitative studies (Chukwuere and Chukwuere, 2024; Huo and Siau, 2023; Kohnke et al., 2024) focus on teachers and students there is a gap in the context of GenAI and its further emergence in the workplace. Characteristics such as the risk of hallucinated outcomes, bias, misinformation, legal implications or other errors (D’Onofrio, 2024; Manduchi et al., 2024; Nah et al., 2023) might present new factors that could contribute to the technostress of workers. At the same time, technological innovations can also foster techno-eustress: a positive form of stress that can drive personal growth, learning, and enhanced performance (Tarafdar et al., 2019, 2024) which could be leveraged in the context of work. Thus, the use of new digital tools can have both burdensome and stimulating effects. The existing models and questionnaires to assess technostress were developed before the introduction of GenAI, so a more comprehensive consideration of this phenomenon is required. It is argued that the current technostress models are no longer sufficient to provide a complete picture (Kumar, 2024) and that there are other contributing stress factors created or enhanced through GenAI (Wach et al., 2023). This is particularly relevant when considering young professionals in industries that will likely experience rapid change driven by GenAI. Many entry-level tasks are highly automatable. Thus, GenAI might intensify concerns in younger cohorts about job displacement, less entry-level jobs and long-term employability (Chui et al., 2023; Ellingrud et al., 2023; Jung and Desikan, 2024; World Economic Forum, 2025). Therefore, young professionals, defined as individuals early in their careers with high adaptability but limited job tenure, are a key demographic to study due to their dual role as both beneficiaries and challengers of emerging technologies (Lattuch and Young, 2011). Furthermore, Lattuch and Young (2011) find that for young professionals the frequency and planning of change are strongly linked to psychological uncertainty, while suggesting that young professionals are comparatively open to change. In the context of GenAI, this combination of change openness and uncertainty sensitivity makes young professionals a particularly relevant cohort for studying GenAI-related technostress. Taken together, young professionals are early adopters who can leverage GenAI for productivity and learning but also a group facing heightened substitution and adaptation pressures. Mapping the current state of the target group could provide additional crucial insights for future generations to determine where support can be offered to enable workers to use and benefit from GenAI in the workplace while minimizing technostress. Building on these considerations, this study poses the following research questions:

1. How do young professionals perceive technostress when using generative AI in the workplace?
2. Which potential new stressors emerge and which are enhanced in the context of generative AI?

By addressing these questions, this study aims to contribute to the ongoing discourse on GenAI’s impact on the labor market, while enriching the technostress literature with empirically grounded insights into GenAI-specific stressors. These findings are particularly relevant for young professionals who will navigate and shape the future workplace, underscoring the need for an early and nuanced understanding of both the challenges and opportunities presented by GenAI.

## 2 Theoretical background

Recent years have marked a significant shift in the dynamics of technological development, largely attributable to the adoption of GenAI. Technologies such as ChatGPT have not only gained importance but have also precipitated a fundamental change in the manner in which work is conducted within organizations. ICT are often defined as the entirety of all technical media used to process information and support communication, including computer and network hardware as well as associated software (Eurostat, 2023). In contrast, the term GenAI refers to a new type of AI-based system that increasingly supports and automates creative and strategic processes (Feuerriegel et al., 2024). However, people are also experiencing new forms of stress, particularly due to the increasing integration of AI into various work processes (Cadieux et al., 2021; Kumar et al., 2024).

### 2.1 Negative impact of technology

The increased use of information and communication technologies (ICT) in organizations is a phenomenon that is now pervasive. This is accompanied by new demands and frameworks, including constant availability, regular updates that need to be implemented and the simultaneous handling of multiple tasks (Srivastava et al., 2015). These developments have been associated with an increased prevalence of psychological stress reactions among employees, which can manifest as feelings of overwhelm (Atanasoff and Venable, 2017; Srivastava et al., 2015). This issue is encapsulated by the term “technostress,” which is defined as “a modern disease of adaptation caused by an inability to cope with the new computer technologies in a healthy manner” (Brod, 1984 p. 16). Although the term has been in use since the 1980s, research in this area is still relatively young. For example, stress resulting from the use of technology in the workplace can negatively impact productivity and performance, leading to high absenteeism and staff turnover, as well as affecting the health of employees (Chen et al., 2022; Daniel, 2019). The increasing volume of information disseminated daily via technology channels such as email, chat and social media, coupled with growing technical complexity, creates a potential for overload that can exceed human processing capacity (Tarafdar et al., 2007, 2011). Furthermore, new technologies accelerate work processes in companies, thereby increasing time pressure (Tarigan et al., 2023). Changes in individuals’ tasks within organisations due to the use of information and communication technologies can also lead to a restructuring of processes within the company (Koraca, 2021). Additionally, the boundaries between professional and private life can become blurred as a result of using digital technologies. This can make employees feel obliged to be available outside of working hours (Konca, 2022). Understanding these effects is therefore essential to develop targeted countermeasures and provide employees with the information they need to reduce ICT-related stress. For instance, Tarafdar et al. (2007) identified five dimensions that can lead to technostress in the workplace: Techno-insecurity (the threat of job loss due to technological advancement), techno-uncertainty (the constant state of change and upgrades in the ICT sector, causing feelings of uncertainty among users), techno-overload (the compulsion to work faster and longer), techno-complexity (the feeling of being inadequate in terms of skills, which forces individuals to spend time and effort learning), and techno-invasion (the obligation to be available at all times, resulting in the indistinguishability of work-related and personal contexts).

### 2.2 Positive impact of technology

While much of the research on technostress has focused on negative stressors, recent studies have begun to pay more attention to positive stressors. These positive stressors emphasise that people can perceive ICTs as challenging and exciting in a positive way, potentially increasing their motivation and performance (Tarafdar et al., 2019). A study conducted by Schlude et al. (2024) concludes that professional users perceive the benefits of GenAI as mostly positive. More than 60% of them reported that the results obtained from GenAI effectively saved time and provided helpful support for challenging tasks. In addition, the use of GenAI can inspire new ideas. Tarafdar et al. (2024) identify four distinct dimensions: techno-mastery, techno-autonomy, techno-relatedness, and techno-enrichment. Techno-mastery describes situations in which technologies challenge and motivate employees to build competence and work more efficiently. Techno-autonomy describes how technology can empower users to act with greater autonomy when prioritizing and executing their tasks. Techno-relatedness reflects how digital tools enable connection with colleagues, facilitating feedback and social support. Techno-enrichment refers to experiences in which technology makes work more interesting and meaningful by helping employees solve problems or create additional value (Tarafdar et al., 2024).

Other studies also indicate that GenAI can have similar positive effects. For instance, Cubillos et al. (2025) report an increased sense of autonomy through the use of GenAI, while Yilmaz and Yilmaz (2023) found enhanced programming self-efficacy and learning motivation. These findings support the dimensions of techno-mastery, techno-autonomy and techno-enrichment proposed by Tarafdar et al. (2024). Both studies were conducted with students learning programming skills. Furthermore, Barana et al. (2023) demonstrate that GenAI can support problem-solving strategies and critical thinking processes. Students evaluated the correctness of AI-generated solutions or verified their own solutions with the help of ChatGPT, leading them to discover new and diverse approaches to problem-solving. These findings highlight the relevance of techno-enrichment, as the use of AI makes work more engaging and enriches learning processes. Finally, Sung et al. (2025) show that AI-supported feedback can enhance learners’ self-efficacy, sense of belonging, and help prevent burnout. In their study, GenAI was employed to improve the practice of periodic feedback by providing personalized, affectively motivating responses in a digital manufacturing course. This illustrates that GenAI can have positive impacts on social aspects, similarly to the techno-relatedness dimension of Tarafdar et al. (2024), which emphasizes connection with colleagues.

## 3 Research approach

In light of the progressively significant role of GenAI in organizational strategies and work processes, it is essential to examine the impact of this technology on well-being, particularly in relation to technostress. While previous studies have examined technostress and GenAI in an educational context (Chukwuere and Chukwuere, 2024; Issa et al., 2024; Huo and Siau, 2023; Kohnke, 2024; Kohnke et al., 2024), there is a lack of research exploring the unique challenges posed by GenAI technologies in the workplace. Wach et al. (2023) already suggest a link between GenAI and technostress at work, but without providing in-depth empirical evidence. In light of this paradigm shift the question arises as to whether the traditional definition of ICT, as employed in established technostress scales, remains adequate to capture the challenges emerging from GenAI. In consideration of this context, a qualitative research design was chosen to capture in-depth insights into this emerging phenomenon. A total of 15 young professionals between the ages of 26 and 35, with a maximum of 5 years of work experience and at least a bachelor’s degree, were recruited for the study. The respondents’ professional backgrounds are as follows: four respondents are employed in the field of IT, four respondents in R&D, two respondents in the field of finance, and five respondents in marketing (Table 1). They were contacted through professional networks and referrals within organizations that had either piloted or adopted GenAI.

**Table 1:** Distribution of interviewees and their stance to the established stressors.

ID	Industry	Exp	Techno-insecurity	Techno-uncertainty	Techno-overload	Techno-complexity	Techno-invasion
R&D1	Research & development	3.5	Reject	Neutral	Neutral	Reject	Reject
R&D2	Research & development	1.5	Reject	Neutral	Neutral	Agree	Reject
R&D3	Research & development	2	Neutral	Agree	Neutral	Agree	Neutral
R&D4	Research & development	3	Reject	Agree	Agree	Agree	Agree
IT5	Information technology	1.5	Reject	Agree	Reject	Agree	Agree
IT6	Information technology	2.5	Reject	Reject	Neutral	Agree	Neutral
IT7	Information technology	2	Reject	Agree	Agree	Reject	Neutral
IT8	Information technology	2.5	Reject	Neutral	Agree	Reject	Reject
FIN9	Finance	5	Reject	Reject	Agree	Reject	Agree
FIN10	Finance	2	Reject	Neutral	Reject	Reject	Agree
MA11	Marketing	4	Reject	Reject	Agree	Reject	Reject
MA12	Marketing	2.5	Reject	Reject	Reject	Reject	Reject
MA13	Marketing	3.5	Reject	Reject	Reject	Reject	Reject
MA14	Marketing	5	Reject	Reject	Reject	Neutral	Neutral
MA15	Marketing	4	Neutral	Reject	Reject	Neutral	Reject

The semi-structured interviews were conducted using the approach of Bogner et al. (2014). The interview guide combined a structured, deductive core grounding in known technostress dimensions (Tarafdar et al., 2007, 2019, 2024) with open-ended questions that allowed individual experiences and unexpected insights to emerge. The development of the interview questions followed a deductive approach in accordance with the principles of qualitative content analysis as outlined by Mayring and Fenzl (2019). The initial set of questions explored the five established technostress dimensions: techno-insecurity, techno-uncertainty, techno-complexity, techno-invasion, and techno-overload (Tarafdar et al., 2007). To this end, questions were developed deductively based on Tarafdar et al.’ dimensions in order to obtain more comprehensive information and gain insight into the participants’ attitudes towards GenAI. Examples of guiding questions included: “Do you feel that GenAI poses a threat to your role or job security?” (techno-insecurity) or „When using GenAI tools at work, how easy or difficult do you find it to understand and use them effectively? “(techno-complexity). To capture additional GenAI-related challenges (D’Onofrio, 2024; Manduchi et al., 2024; Nah et al., 2023), we deductively designed further questions. These questions were also based on past studies that had investigated other aspects of technostress, such as the unreliability of ICT systems (Ayyagari et al., 2011; Califf et al., 2020) and uncertainty about compliance (D’Arcy et al., 2014; Hwang and Cha, 2018), both of which are challenges posed by GenAI. The goal was to identify new organizational and individual effects of the implementation and use of GenAI. Examples of these questions contained: Thinking about your everyday work: What has changed for you since you started using GenAI tools like ChatGPT or similar applications?,” “How do you typically deal with the outputs GenAI provides? What’s your process for deciding whether to trust or adjust them?” or “In what ways, if any, has GenAI influenced how you develop your skills or think about your own expertise?.” The full interview guidelines can be found in the Supplementary material.

Each interview lasted between 35 and 48 min. All interviews were audio-recorded, transcribed, and analyzed using structured qualitative content analysis (Mayring and Fenzl, 2019) in a two-stage process using MAXQDA 2020 (20.4.2). The responses of the first set were analyzed using deductive category application, assigning segments of text to the corresponding predefined categories. In addition, the analysis also included a second layer of interpretation: for each of the five established categories, the researchers subjectively and hermeneutically derived whether the participant’s response indicated agreement, rejection, or a neutral stance toward the respective stressor. This interpretive judgment was made based on the content, tone, and framing of the participants’ statements. Further in this process, aspects of the second set or those that could not be categorized within the traditional dimensions were also coded and categorized. This enabled new challenges and problem areas in connection with GenAI to be identified. In the second round, all authors collaboratively reviewed and refined these codes. This joint review and refinement process ensured the consistency and reliability of the coding scheme. Building on the work of Guest et al. (2006), who demonstrated that approximately 12 interviews already capture about 97% of thematic content, we observed thematic saturation on the subcodes after 11 interviews. Given our scope and sample homogeneity, a sample of 15 interviews is appropriate. Nevertheless, due to limited subsamples, especially in finance, sectoral patterns should be interpreted as exploratory.

## 4 Results

**Figure 1:**
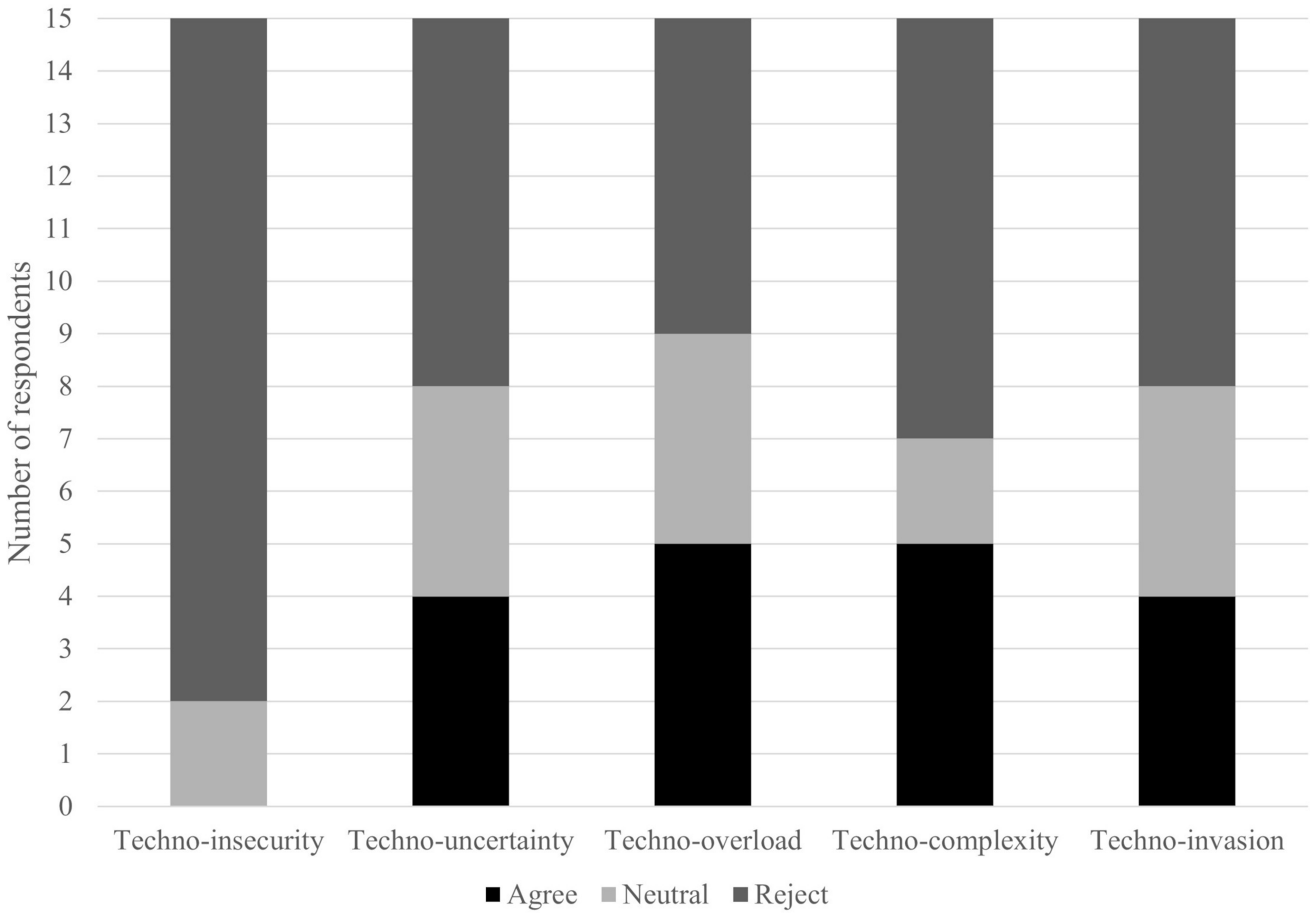
Responses on established stressors.

This section presents the results of the interviews, disaggregated and discussed by industry sector. The analysis differentiates between established techno-distress dimensions established in prior research (Tarafdar et al., 2007) positive stressors and new knowledge that has been less researched and established to date. Figure 1 displays the response distribution on the five established stressors: “Agree” indicates that participants perceive the stressor, whereas “Reject” indicates they do not. The exact distribution of opinions on these stressors among the interviewees and their respective sectors can be found in Table 1.

An additional 155 codes overall emerged from the data on other challenges, as well as other positive aspects regarding novel or unexpected concerns (Table 2). A full codebook with additional descriptions and anchor quotes can be found in the Supplementary material.

**Table 2:** Distribution of categories and subcodes.

Positive Stress	35	Reliability	31	Cognitive effects	36	Compliance	33	Role Shift	17
Work Simplification	14	Control and verification	14	Dependency	12	Data security	13	Job change	8
Motivation	10	Prone to errors	9	Skill degradation	8	Copyright	6	Multi-Level-responsibility	5
Enjoyment	6	Hallucination	4	Creativity	7	Regulation	5	Process problems	3
Technology Affinity	5	Bias	4	Learning experiences	5	Trust	5		
				Critical thinking	4	Ethics	4		

### 4.1 Techno-insecurity

Almost all participants, regardless of industry, do not see their jobs directly threatened by GenAI. However, there is a general sense that GenAI will lead to significant changes in job roles and areas of work in the future. Many participants emphasized that, despite the rapid technological advances in AI, human supervision, contextual understanding and critical judgement will remain indispensable. As R&D4 explains: *“It quickly becomes apparent that AI tools have their limitations. A human component is still required, even for simpler tasks.* Similarly, MA12 confirmed: *„Putting that emotional aspect into it and all that background information is something AI just cannot do in the same way. It does not know how it all fits together, and that’s one of those things where I know it will never be able to replace me. “*FIN10, added: *„So now, in the field of auditing, it will never be able to replace the work of the auditor.”* While the respondents largely rejected the idea of a complete replacement of human work by GenAI, but emphasized its transformative effect on workflows, responsibilities, expectations and organizational change. IT6 put it this way: *“I do think the industry will change somewhat. But it’s not like people could just be replaced.”* MA14 further elaborated: “*Some jobs will die out. I used to work a lot with transcription agencies in market research, and they are facing tough times because they will become relatively redundant relatively quickly if things continue to develop in this way.”* MA15 also expresses a certain frustration that the computer can complete tasks much faster than they can: *“It’s difficult to break away from that. Now that I’ve generated an image, I’m suddenly supposed to create something of my own without copying what’s already there. That could be difficult, and I imagine it’s very frustrating when a computer is simply much faster than I am. When I imagine that it takes me eight hours to create a commercial and then a computer does it in two minutes, that can be stressful.”* Finally, participants noted that media coverage can create uncertainty and stress about potential job losses. R&D3 elaborated: *“Articles about new GenAI models or how they might replace programmers can make you question your own skills. That can be stressful.”*

### 4.2 Techno-uncertainty

The perception of uncertainties associated with the rapid changes and developments brought about by GenAI varies among the respondents. In particular, participants from R&D describe a pronounced sense that the technology is evolving at high speed and that new, more powerful models are entering the market at short intervals. This is described as a source of uncertainty and simultaneously creates the impression that one must continuously stay up to date. R&D2 expresses this as follows: *“I would say that, basically, I find it rather difficult to keep up with what’s going on and where progress is headed, because so much is changing. So you have to keep up with it a little bit all the time to avoid being left behind.”* R&D3 describes a similar feeling*: “Yes, I do. Partly out of interest, partly because I feel like I have to keep up. It feels like a new model comes out every two weeks that is supposedly better, faster, and more powerful. Then you have to ask yourself how you can use it*. “IT5 articulates the fears associated with this dynamic even more clearly: *“I’m a little scared because the development of AI is continuing so rapidly. But I always try to stay positive. You always have to overcome new challenges.”* In contrast, respondents from the marketing and finance sectors largely express no concerns. Only FIN9 makes a critical comment: *“It’s difficult to stay up to date all the time.”*

### 4.3 Techno-overload

Assessments of the effects of GenAI tools, such as ChatGPT, on workload and working hours vary considerably. Respondents from marketing predominantly report positive effects. However, MA11, who otherwise emphasizes the advantages, voices a reservation: *“As a result, everything becomes even more compressed, I do believe that.”* This perception is reinforced by FIN9 and explicitly points to the issue of techno-overload. He notes: *“These new technologies have always been introduced with the promise of making everything easier. In fact, however, something else has always materialized, namely, the pace has increased enormously and accordingly, so has the stress.”* In addition, almost all of the respondents from R&D indicated that the proportion of complex tasks is steadily increasing, while simpler tasks are becoming less frequent. Consequently, the total number of work tasks is also rising. R&D4 explains: *“If we finish faster because of AI, we might get more assignments and in the end, face more pressure because the quality still has to be maintained. Those who are skilled in using AI complete their tasks more quickly. This can generate additional pressure.”* In this context, an increase in work intensity is also observed. R&D2 emphasizes: *“In the past, smaller tasks also provided a kind of mental break. Now it’s only complex tasks. If I’m only doing complex tasks, at some point my head just says ‘no.’ If I spend an entire day working on highly conceptual tasks with hardly any simple ones, I come home after work and cannot really think anymore.”* Furthermore, IT7 stresses that the use of AI leads to stronger parallelization of activities, which may result in overload: “*One person could be involved in many more topics simultaneously if AI takes over parts of the programming. This can lead to a form of overburdening. If, as a human, I am primarily monitoring instead of programming myself, then I might have ten projects simultaneously and no longer this ‘straightforward’ spectrum of tasks.”*

### 4.4 Techno-complexity

Respondents’ assessments of the extent to which the complexity of GenAI constitutes a form of overload, complicating the acquisition, understanding and effective use of these technologies, vary considerably. Around two-thirds of respondents from marketing currently see little need to engage with GenAI in greater depth, as these technologies have played only a minor role in their organizations thus far. However, MA14 explicitly emphasizes that systematic engagement with GenAI will be indispensable in the future to remain competitive: *“We also see that there are competitors who are already much further ahead. You can already notice that at some point someone has a GenAI solution in the advertising or media sector, and you think: Okay, we could not do that at all, because we do not even know how it works. We would not know whom to ask, how to get started, or what would be required for it. There is definitely a large gap.”* MA15 likewise highlights the challenge posed by the growing complexity of GenAI. A similar perspective is shared by R&D2, R&D3, R&D4, IT5 and IT6. They all stress that working with GenAI requires in-depth expertise, and that the constant emergence of new developments fosters a fear of not being able to keep pace. IT5 articulates this as follows: *“Sometimes I am afraid because something new comes up every day. You constantly have to overcome new challenges. That’s also the case with ChatGPT. At work or after work, you always have to search Google or the internet and find new information.”* Those involved in R&D view the acquisition of deeper knowledge as indispensable. For example R&D4 explains: *“We conduct a lot of research in the medical field. You really need to know how the model was trained, and that makes it very complicated.”* However, R8 also emphasizes: *“For many people, it is sufficient to use the basic version.”*

### 4.5 Techno-invasion

The majority of respondents from the R&D sector reported using GenAI tools in their personal time. However, the extent to which this was perceived as pressure, particularly due to concerns about falling behind professionally, varied considerably. Around half of the participants said they engaged with the technology primarily out of personal interest, while others said they had no opportunity to do so during working hours. R&D4, for instance, stated: *“I cannot simply take a day off each week to follow the latest developments. This already creates a certain pressure to engage with it in my free time. Regarding quality: if someone with little experience polishes up code, this could increase the pressure to use the tool as well in order to keep up.”* Similarity, IT5 noted: *“At work or after work, one always needs to research and find new information. I therefore constantly try to improve my knowledge. After work, I often use ChatGPT. In general, I believe it is important to engage with new technologies in my professional environment in order to remain relevant in the job market.”* Finally, IT6 highlighted that, given the ongoing changes in the industry, private learning may become increasingly necessary: *“In the future, if the industry changes significantly, one would probably need to pursue additional training in private time. I can understand that this creates pressure for some people. The feeling that one must educate oneself now because there is no time to do so at work in order to remain competitive or progress professionally.”* Participants from the finance sector expressed similar perspectives. As FIN10 explained: *“Either you keep up with the times, or you do not. Depending on how skilled you want to be, you also have to engage with it in your free time. Because during working hours, we have a relatively high workload. There’s no time for it then either.”* Participants from marketing did not report any negative concerns regarding the use of GenAI.

### 4.6 Further findings

Positive stress: While much of the discussion centered on negative technostress and related challenges, the interviews also revealed the consistent presence of positive stressors, particularly those associated with intrinsic motivation, perceived efficiency gains, enhanced performance, and the generation of new ideas. Notably, however, participants from the finance sector reported comparatively fewer positive stressors than those from R&D or marketing. MA12 for example added: *“For us, it is more of a benefit than a burden. Generating the same output with less input. It is a useful tool. Our work is becoming increasingly complex. Through ChatGPT we are able to manage this workload.”*

Respondents working in R&D and IT particularly emphasized how GenAI enhances efficiency and overall performance. R&D3 and IT5 also stressed this point: *“You no longer have to write every single piece of code yourself and can instead focus on more complex tasks”* (R&D3), and *“If you know how to use ChatGPT well, you can quickly get your task done. I think that’s great”* (IT5). Participants from the marketing sector expressed similar views. MA11 emphasized: *“I think it is more supportive. It makes many things easier, so I rather believe in the positive effects in the work environment.”* MA12 likewise highlighted efficiency and time savings but noted that his employer was not yet fully aware of these benefits: *“My employer partly does not even know how much efficiency it brings me, so I am given more time than I actually need in theory.”* He further added that it is *“very smart to use it in one’s free time as well, simply because it really saves an incredible amount of time.”*

Another aspect that was frequently highlighted, particularly by respondents from R&D and IT, is the personal interest and intrinsic motivation in engaging with GenAI. R&D2 stated: *“I would not say there is a lot of pressure that I absolutely have to do this to keep up at work. I rather have a certain intrinsic motivation.”* IT7 also emphasized that he enjoys trying out new things: *“I enjoy being able to test new things. I am not overwhelmed, but rather interested. I do not see this as a challenge at all, but rather have fun with it.”* R&D1 was even more specific and related his interest to image generation: *“So I have spent a lot of time working with image models, because it is not relevant for work and I just found it interesting.”* In addition, a respondent from marketing also reported a certain curiosity: *“I bring a basic curiosity with me which for me basically means that I say I want to know how does this work or how is this done? Just this sense of trying things out”* (MA15).

Another positive aspect, mentioned exclusively by participants from marketing, concerns idea generation. MA13 explained it as follows: *“Everything goes much faster; idea generation or information acquisition; knowledge expansion; you can generate multiple answers to the same question if you are not satisfied.”* MA14 also emphasized this advantage: *“We knew we needed a lot for dynamic content and just said: give me 50 headlines for this or that topic and that takes only 20 s, whereas it would otherwise always take half an hour.”*

In addition to these stressors, the interviews also revealed other concerns, which are clustered below.

Compliance: The most frequently cited concerns relate to compliance, particularly with regard to ethics, regulation, copyright, and data protection. FIN10, raised ethical reservations but did not elaborate further: *“I personally find that in the area of ethics we really have to be careful. The technology is quite new and evolving rapidly. I’m optimistic, but still rather skeptical.”* Detailed responses concerning the lack of regulation were provided by several participants. IT8 emphasized the importance of regulatory frameworks for the use of GenAI: *“Of course, with constant progress new regulations always need to be created, otherwise theoretically you could do anything with an AI, which would not necessarily be in line with the law.”* R&D1 went even further, stressing the lack of transparency: *“We have absolutely no guidelines for official use. And I have the feeling that either people ‘over-use’ it, or on the other hand do not really know what they could actually do with it.”* R&D3 and MA11 supported this assessment. As R&D3 noted: *“It’s really critical when private accounts are used for official purposes. But there are no clear rules. I’d prefer if there were very concrete regulations.”* Similarly, MA11 remarked: *“I know the risks are already enormous. I hope this will somehow be contained or kept in check through regulation.”* MA14, connected the absence of regulation to copyright issues and highlighted the uncertainty regarding the originality of content: *“This whole topic of who actually owns which idea or so as if we were now starting to copy things and had never done that before,that’s nonsense. We’ve always freely borrowed ideas, but it used to be a bit easier to determine: does this belong to me or not? Now there’s this added uncertainty. We also hand things over to others. When is my idea really mine? That gets heavily blurred with all these GenAI platforms.”* He further stressed the lack of transparency about data usage: *“When Meta reserves the right to grab the data in order to build its own AI models with it. Against this background, I find it extremely problematic how little is being controlled and regulated.”* The most frequently mentioned concern, particularly among participants from R&D and marketing, was data protection. R&D1 illustrated this with a practical example: *“It already starts with simple things like ‘let us write a letter’ and then people just copy in all the information.”* R&D4 also emphasized the increasing complexity of data protection: *“Data protection is becoming more and more complex, especially because of the AI Act, Data Act, GDPR, DIPA, DiGA, and so on. We are facing very strict restrictions, particularly when it comes to sensitive patient data.”* MA14 confirms this, while acknowledging his own lack of expertise in the field: *“Definitely not uncritical. I’m not the biggest data expert when it comes to this kind of thing, so I think sometimes I personally approach it a bit naively. That’s why it’s definitely a critical area where, I think, people who work with it really need to develop awareness.”* For these reasons, MA11 explained that he hardly uses GenAI in private life: *“I do not really use it privately either, partly to protect myself and partly for data protection reasons. Because I think, theoretically, you could have everything recorded all day long, or rather everything is always listening, and I really do not want that because it does not give me a good feeling.”*

Cognitive Effects: A significant number of respondents from R&D and marketing also express concerns about long-term cognitive consequences. In particular, they emphasized the potential emergence of dependencies, which could affect the way knowledge is acquired and processed. R&D1 describes a shift in the process of knowledge acquisition and points out that the technology has influenced the competence development: *“If this technology had not come, I would probably have acquired significantly more skills in programming.”* At the same time, R&D1 acknowledges a dependency associated with its use: *“if the tools were to suddenly disappear, then I would probably be worried. Then I would have to relearn all that.”* Nevertheless, it is emphasized that this does not necessarily lead to a general loss of skills, but rather to a shift: *“So it is more like I lack skills that I would otherwise have learned, but instead we acquire other skills, in this case in dealing with GenAI.”* Similar tendencies are described by R&D3, who implicitly notes a deterioration in the skillset: *“I have definitely gotten worse because I already rely on the AI to take over part of the work for me.”* R&D4 explicitly points to the danger of convenience: *“Dependencies can arise because it is convenient.”* and warns of the need for reflective use: *“One can certainly learn from ChatGPT if one understands it only as a tool. But if one believes that the AI takes everything off one’s hands, one shoots oneself in the foot, because that does not work.”*

The potential influence on cognitive processes also becomes visible in the marketing sector. MA13 emphasizes that, through the use of AI, it has to engage in less independent thinking and linking: *“Simply not having to think about things again” and that one “also does not have to have all the connections in one’s head.”* In addition, MA13 stresses a decline in creative performance*: “one somehow under-challenges oneself a bit when one or others use artificial intelligence and as a result I believe creativity already shrinks a little bit.”* Another critical aspect is highlighted by MA14, who points to possible losses of knowledge in the evaluation of advertising effectiveness studies. He describes that individuals without relevant expertise generate results without understanding their methodological basis: *“I see it as a bit critical with us, because with us advertising effectiveness is sometimes evaluated with ChatGPT, that you then say, well, we have an advertising effectiveness study here and we throw an Excel table into ChatGPT and then say please tell me if you apply this test? We have people who did not study this, who do not know what they are interpreting, who throw a table in somewhere a number comes out at the bottom and then they say okay, I have now found my truth.”* MA15 also emphasizes a certain dependence on GenAI: *“If AI is responsible for processes or is held responsible, then there is definitely a certain degree of dependence.”*

Reliability: A critical aspect of using GenAI is their controllability and reliability, a point emphasized particularly by respondents from marketing, R&D and IT. R&D1 explains that using such models can increase the maintenance effort in software development: *“What I see as the bigger problem is that you definitely need to carefully control what actually comes out. I suspect that this ultimately results in a huge maintenance effort because no one on-site properly documents and checks this code if needed. In the worst case, the code does not do what it is supposed to, and no one notices, producing errors. Then you have to extend it further without knowing what happened back then, what a GenAI model wrote, and you completely lose oversight.”* Similar concerns were expressed by R&D4 and IT5. R&D4 points out that AI tools can generate additional workload due to their high accessibility, and they may also create unrealistic expectations: *“ChatGPT is so accessible and convinces many people who do not understand how it works in the background. Then, as a developer, you need to invest a lot of effort to communicate realistic limits. AI tools can sometimes slow down workflows, especially because you become more aware of their possibilities. ‘I could also run this through DeepL or ChatGPT’. That, in turn, takes additional time. Once you realize that much more is possible, you want to invest more time to get the optimum. So, there is a new kind of pressure.”* IT5 criticizes the lack of consistency in outputs, which limits trust in the results: *“When using ChatGPT, sometimes there are different answers, and that bothers me because I cannot rely on it.”* Respondents from marketing also emphasized the importance of critically reviewing AI-generated content. MA12 points to potentially inaccurate information: *“You always have to be careful about the information you receive, as the texts are not always entirely correct.”* MA13 stresses that uncritically adopting such content is problematic, and that domain expertise remains essential: *“Especially when it eventually becomes specific content from ChatGPT and you can no longer trace whether it is correct; your own skills in this area remain absolutely necessary.”*

Role shift: The majority of respondents emphasize that the way work is conducted in organizations is likely to change as a result of the increasing implementation of GenAI. Particularly, participants from R&D and IT anticipate that job profiles and task contents will need to be adapted. IT6 remarks: *“Well, I do believe that the industry will change a bit. But it’s not as if you could be replaced. I can imagine that a simple software development job will not make much sense anymore. Maybe you’d have to get further training.”* Similarly, IT7 describes that tasks will not only change but may also become more complex: *“It will simply change the work, but there will be new tasks, such as sensible prompt design. I am not afraid of being completely replaced by AI. If, as a human being, I am more likely to just supervise rather than program myself, I will have 10 projects at the same time and no longer this ‘straightforward’ range of tasks.”* R&D2 further emphasizes that it is not only the execution by systems that is crucial but especially the planning and conceptualization of systems: *“It’s not just ‘computers telling him what to do’ that the systems are good at, but also designing systems and coming up with an approach that needs to be thought through. Ultimately, as you said, it boils down to something more conceptual.”* A similar perspective is shared by participants from the marketing domain. MA11 points out: *“I believe it will change many areas of work. We already use AI extensively in the field of graphics to generate images or even for inspiration in text. It’s not yet at a very high level, but it’s definitely always a good foundation.”* MA12 adds: *“It will not do it completely on its own, so it needs me to do some of the work.”* Some respondents see responsibility for implementing and use of AI primarily at the organizational level. MA14 notes: *“Not on a personal level, it’s not personal stress, but I think it’s more on a company level,”* and emphasizes that *“it’s important that this competence is simply available somewhere in a collective.”* Finally, FIN9 highlights potential societal implications: *“Many studies suggest that AI will lead to greater division within society. Some will benefit greatly, while others will come under pressure.”*

## 5 Discussion

Our findings contribute to the existing body of knowledge on technostress by extending the established framework of technostress dimensions (Tarafdar et al., 2007) to the domain of GenAI in workplace settings. While prior research has primarily examined technostress and GenAI in educational settings (Chukwuere and Chukwuere, 2024; Huo and Siau, 2023; Issa et al., 2024; Kohnke et al., 2024) or on management level regarding AI and machine learning integration (Kumar et al., 2024), this study provides empirical insights into the specific challenges that young professionals in R&D, IT, finance and marketing encounter when integrating GenAI into their daily work.

### 5.1 Theoretical implications

Regarding RQ1, the study confirms that young professionals in the examined industries experience established technostressors (Tarafdar et al., 2007) through the use of GenAI to varying degrees across different dimensions. The results highlight the dynamic nature of technostress in the GenAI era and suggest that the manifestation and perceptions of the established stressors in relation to GenAI may differ across industries among young professionals. With respect to techno-insecurity, most participants indicated that their jobs are not directly threatened by AI. However, the majority perceive a transformational change due to AI: tasks and workflows are evolving, while the role of employees remains indispensable. It is also emphasized that media reports on potential job losses can trigger uncertainty and stress among employees.

Participants’ attitudes toward techno-uncertainty differ between industries: employees in R&D and IT report high pressure to constantly keep up and acquire new knowledge. In contrast, individuals in marketing and finance perceive little pressure to adapt. At the same time, R&D employees indicate that, while they are intrinsically motivated to engage with technological changes, this can also lead to stress and strain. These differences illustrate that the perception of technological dynamics is highly context-dependent. In research- and development-oriented areas, feelings of uncertainty and adaptation pressure dominate, whereas other business areas, such as marketing and finance, show comparatively less concern.

Differences also emerge regarding techno-overload. While marketing employees predominantly perceive positive effects, individuals in R&D and finance report higher levels of strain. Work pace increases, simple tasks are eliminated, and complex tasks grow. This accelerated pace also generates additional quality and performance pressure, as multiple projects can be handled simultaneously. On one hand, this can be experienced as motivating and fulfilling; on the other hand, it represents an additional cognitive burden.

Concerning techno-complexity, opinions also diverge. The majority of respondents confirmed that engagement with GenAI may be required during personal time, highlighting the tension between intrinsic interest, professional necessity, and potential pressure. R&D, IT and finance employees report a high need for knowledge to keep up with constantly emerging developments and indicate that knowledge acquisition outside of work hours is necessary. Marketing employees, by contrast, mostly perceive no need to engage intensively with GenAI, as these technologies currently play a minor role in their organizations.

Techno-invasion shows that most R&D, IT and finance employees use GenAI during their personal time as well. Many feel obligated to engage with the technology privately and acquire knowledge to remain competitive professionally. Here, both intrinsic motivation and a sense of duty play a role, particularly in light of high workloads. In marketing, such pressure is hardly perceived.

The results of RQ2 led to the identification of other factors that could contribute to technostress. These include compliance-related issues, cognitive effects, reliability, role shift and positive stressors. The set of codes regarding compliance refers to the regulatory and ethical uncertainties accompanying the adoption of GenAI tools. This captures concerns related to data protection, information security, and adherence to organizational guidelines, which previous studies link to technostress (D’Arcy et al., 2014; Hwang and Cha, 2018; Hwang et al., 2022), particular to the dimensions techno-complexity and techno-uncertainty. Moreover, it highlights the evolving regulatory landscape, exemplified by emerging legislative frameworks such as the EU AI Act, which impose new requirements and obligations (European Union, 2024). The issue of compliance is not new to technostress, but it is being amplified by GenAI due to new regulations, copyright laws, and emerging data protection risks. Consequently, GenAI implementation introduces additional compliance challenges that extend beyond conventional IT governance, thereby adding both cognitive and administrative burdens on employees and organizations.

Another issue is the cognitive effect of using GenAI. Many respondents expressed concerns about growing dependency, which could result in a reliance on convenience, reduced individual performance and the risk of using knowledge superficially. A possible decline in creative capacity was also viewed critically. These consequences are supported by studies from Caporusso (2023) and Lee et al. (2025). They demonstrate that the deeper GenAI-based tools are embedded into organizational workflows, the stronger the concerns regarding the potential erosion of human capabilities, such as critical thinking, independent problem-solving, and creativity. This heightened dependence may introduce new strains on individuals, particularly when GenAI tools become indispensable to day-to-day tasks and decision-making processes. These cognitive effects could be stressors that have not yet been fully considered in technostress research and are novel in the context of GenAI. In recent studies (Chen et al., 2025; Gerlich, 2025; Lee et al., 2025; Zhai et al., 2024), these phenomena have been examined in a broader sense, giving rise to an emerging research stream on the cognitive and behavioral consequences of GenAI use.

Conversely, it is emphazised in the interviews that GenAI does not necessarily result in a complete loss of knowledge, but rather a shift in competencies. While traditional skills may lose some of their significance, new AI-specific competencies emerge that can replace or complement them. Overall, a field of tension emerges between the risks of dependency and reduced creativity, and the opportunities of competence shifts and new skills. This is also reflected in the observed role shift and underscores the need to consider the potential social implications of AI implementation within organizations, particularly with regard to evolving role perceptions. The results suggest that GenAI is perceived as a driving factor for profound changes in the world of work. The deployment of AI will not only change existing work content and tasks but also create new areas of responsibility. For the successful integration of generative AI within organizations, the establishment of clear roles and responsibilities is essential. This is particularly important in heterogeneous teams, where employees possess varying levels of experience and expertise with digital technologies (Stowasser, 2025). Changes in task portfolios, uncertainty about future skill requirements and concerns about job security are well-recognized stressors in technology-rich workplaces (Tarafdar et al., 2007; Srivastava et al., 2015). Conceptually, the role shift partly builds on existing technostress constructs such as techno-insecurity and techno-overload. However, our findings suggest that GenAI intensifies and accelerates these stressors. Skill requirements evolve more rapidly, task changes become more widespread, and concerns about future employability are increasingly tied to mastering GenAI-specific competencies.

The last important set highlighted by individuals from marketing and R&D is reliability. The findings underscore the challenges posed by AI-generated errors, hallucinations, inconsistencies in model outputs, and the increased cognitive load needed for quality control and verification. Previous studies have already shown that unreliability in ICT can act as an additional stressor (Ayyagari et al., 2011; Califf et al., 2020; Fischer et al., 2019). Following from that, it is crucial for users to maintain a high level of attention and check the credibility of AI-generated content. This might lead to an increase in stress levels and further contribute to an even more complex work environment, as shown in the context of techno-uncertainty and techno-complexity. Even though the unreliability of technology is already associated with technostress, GenAI opens up new facets of stress through novel aspects such as hallucinated outcomes, verification efforts, and other error-prone behaviors (Manduchi et al., 2024; Nah et al., 2023).

Beyond techno-distress, our findings consistently show positive stressors associated with GenAI use. Many interviewees described how GenAI enabled them to produce the same results with fewer inputs and cope with growing job complexity, perceiving GenAI as a benefit rather than a burden. This aligns with the concept of techno-eustress (Tarafdar et al., 2019, 2024), defined as stress responses that are perceived as challenging rather than hindering and associated with learning and improved performance. Regarding this topic, the interviews reveal connections to established techno-eustress dimensions (Tarafdar et al., 2024). Efficiency gains and the ability to focus on more complex tasks correspond to techno-mastery, where technology supports the development and application of advanced skills. The ability to perform tasks more efficiently and using GenAI to manage a growing workload reflect techno-autonomy, as participants feel more in control of when and how they complete their work. Experiencing intrinsic motivation and enjoyment when experimenting with GenAI, sometimes outside of one’s formal job, reflects techno-enrichment. In this case, technology becomes a source of personal development and stimulation rather than a source of pressure. These observations are further supported by other studies reporting positive effects of GenAI. For instance, Cubillos et al. (2025) found that the use of GenAI increased users’ sense of autonomy, while Yilmaz and Yilmaz (2023) observed enhanced programming self-efficacy and greater learning motivation. In marketing, the emphasis on rapid idea generation and creative support suggests a form of techno-relatedness, where GenAI is perceived as a collaborative partner in creative work rather than a competitor.

Taken together, our results support a dual-path view of GenAI-related technostress among young professionals. The same technology can create both distress and eustress. Whether GenAI is primarily experienced as a hindrance or a challenge appears to be shaped by contextual factors, such as sector, task type and the opportunity to translate efficiency gains into meaningful upskilling. These aspects provide initial insights into how the understanding of experienced technostress in the examined industries can be expanded. Integrating these into existing theoretical frameworks and measurement instruments could enable researchers and practitioners to better anticipate and mitigate new stressors in GenAI-driven work environments.

### 5.2 Practical implications

The insights from this study can be utilized to develop recommendations for practitioners, with the objective of reducing stress and empowering employees to work optimally with GenAI. Regarding compliance, our findings on regulatory uncertainty and data security align with the growing body of research on shadow AI (Silic et al., 2025). To reduce compliance uncertainties and avoid potential data protection violations, the authors recommend providing approved, enterprise-grade GenAI tools. They also suggest issuing short, practical policies that clarify acceptable use and data-handling rules while integrating these policies into targeted AI literacy training (Silic et al., 2025). Recent studies show that the reliability of GenAI, particularly LLMs, remains questionable in terms of hallucinations and other errors. Thus far, there are no fully reliable technical solutions (Huang et al., 2025), so human review remains indispensable. However, while managers often expect employees to work faster with AI (Monahan and Burlacu, 2024), they may underestimate the amount of review and verification work required to obtain reliable results. Therefore, organizations should explicitly plan their verification efforts and allocate time for employees to review AI-generated outputs. The cognitive effects cluster highlights tensions related to dependency, perceived skill degradation, creativity, and critical thinking. According to recent work by Lee et al. (2025), GenAI at work should be used as a scaffold rather than a crutch. Tools and workflows should explicitly promote verification and task stewardship so critical thinking is repurposed rather than replaced. In line with Gerlich (2025), organizations should aim to integrate GenAI in a balanced way. This involves designing tasks and training that require employees to engage with and justify AI-generated content. It also involves fostering awareness of when and how to use GenAI so that it complements core cognitive skills. The role shift cluster shows that GenAI primarily takes over easily standardized tasks and changes jobs by freeing up time for complex activities. Human work might increasingly centre on oversight, interpretation and sense-making, especially in knowledge-intensive industries (Butollo et al., 2025; World Economic Forum and PwC, 2024). Rather than leaving these shifts implicit, organizations should explicitly communicate changing expectations in job descriptions and performance criteria. They should also involve all employees, particularly young professionals as early adopters, in co-designing GenAI-supported workflows and link GenAI adoption to transparent upskilling and reskilling paths (World Economic Forum and PwC, 2024). Recent work on technostress among older workers shows that digital change reshapes intergenerational knowledge flows (Li et al., 2025). Our findings indicate that young professionals, while also experiencing GenAI-related stress, possess critical GenAI know-how. Organizations could leverage this by establishing mentoring and learning formats in which young employees share GenAI practices, while more experienced colleagues contribute domain expertise and professional judgement. On the side of positive stressors, companies should leverage these accordingly. Rather than viewing GenAI solely as a risk, organizations can proactively design for eustress pathways. They can do so by creating safe spaces for experimentation, recognizing GenAI-enabled improvements in performance evaluations and leveraging GenAI to enrich work instead of simply accelerating routine tasks (Tarafdar et al., 2019; Tarafdar et al., 2024; World Economic Forum and PwC, 2024).

Overall, all findings align with the recommendation of training and enhancing AI literacy among all stakeholders in the workplace (Pinski and Benlian, 2024; Söllner et al., 2025), the call for human-centered AI (Söllner et al., 2025), and a flexible, community oriented approach (Cardon et al., 2023). In addition, the Future of Jobs Report indicates that 77% of surveyed employers intend to reskill and upskill their workforce to ensure optimal functionality alongside AI (World Economic Forum, 2025). In the context of the AI Act, this is no longer just a formal recommendation. Instead, as of February 2, 2025, it has become mandatory for companies to ensure that their workforce possesses a sufficient level of AI literacy (European Union, 2024). Initial findings also suggest that technostressors, such as techno-overload, can be effectively reduced through targeted training measures (Illgen et al., 2026). Moreover, it is recommended to establish a structured guideline or governance to provide employees with clear information on the scope and purpose of GenAI use. Such a framework should clearly define the scope and purpose of GenAI use. Raising awareness of issues such as regulation and privacy is key, as these are often perceived as barriers. Additionally, more educational work should be done to make it clear that GenAI is not intended to replace human labor, but rather to act as a supporting tool that promotes productivity and creativity. Therefore, this study comes to the conclusion that dealing with GenAI and the associated challenges is a complex issue that harbours both opportunities and risks. A comprehensive, ongoing examination of these aspects is crucial in order to fully exploit the potential of GenAI while minimizing the negative impact on employees’ mental health and well-being.

### 5.3 Limitations

This study offers valuable insights into the impact of GenAI on technostress among young professionals. However, it is essential to acknowledge the study’s limitations. Firstly, while the sample size was sufficient for qualitative research, it limits the generalizability of the findings to broader populations. The study was conducted with young professionals in industries particularly affected by GenAI, meaning the results may not fully capture the experiences of individuals in other sectors or age groups where GenAI adoption is slower or less disruptive. In addition, the industry distribution was unbalanced and patterns should be considered as exploratory. Although a pre-test was conducted, some open questions appear suggestively worded, so response bias cannot be entirely ruled out. Furthermore, notwithstanding the authors’ interviewing experience and the use of follow-up questions to mitigate interviewer bias, a residual risk of influence on the results remains.

Despite these limitations, the study makes an important contribution by extending existing technostress frameworks and identifying novel GenAI-specific stressors. It should also be discussed whether emerging stressors should be regarded as an independent construct or rather as a supplement to the existing technostress constructs. The definitions and items for measuring the established technostress dimensions could be adapted accordingly. The study’s findings lay a solid foundation for future research, particularly in the development of updated measurement tools and intervention strategies to enhance understanding and management of the challenges posed by GenAI in professional settings.

## 6 Conclusion

The presented study highlights the transformation of technostress in everyday working life of young professionals in R&D, IT marketing and finance sectors through the emergence of GenAI and shows that traditional stressors are still relevant, but new aspects have gained relevance. The results underline the productivity-enhancing and innovative potential of GenAI tools. However, they also highlight that existing technostress models might need to be adapted to reflect the complexities of GenAI integration in the workplace. Although many professionals recognize the potential benefits, concerns about regulatory uncertainty, increasing reliance on GenAI and the quality of GenAI-generated results suggest that organizations need to provide clear guidance and support to help workers overcome these challenges and offer proactive strategies. Given the findings of this study regarding current technostress measurement models, a future direction is the development and validation of an updated questionnaire that integrates the recently identified stressors specific to GenAI. A key aspect should be a revision of the established stressors and the consideration of GenAI-specific factors. An examination of industry-specific variations would also provide insights into whether certain professions experience these challenges more acutely than others. Further studies could investigate how organizations can develop interventions to mitigate negative stressors while promoting the beneficial potential of GenAI. Furthermore, measures for the reduction of negative stress and the promotion of positive stress should be reviewed. Finally, as regulatory frameworks around GenAI continue to develop, research into effective governance and policy measures will be crucial in ensuring that AI adoption aligns with both organizational goals and employee well-being. Addressing these areas will contribute to a better understanding of the balance between the opportunities and risks of GenAI. Ultimately, this will contribute to a more sustainable and adaptive integration of AI in the workplace.

## Data Availability

The original contributions presented in the study are included in the article/Supplementary material, further inquiries can be directed to the corresponding author.
