# Supplementary material for: Technostress and generative AI in the workplace: a qualitative analysis of young professionals
Source: Front Artif Intell. 2025 Dec 12;8:1728881. doi: 10.3389/frai.2025.1728881 (PMC12741148; doi:10.3389/frai.2025.1728881)
Supplement: Supplementary file 1 [file Data_Sheet_1.pdf]

Interview Guidelines

1. Techno-Insecurity *(Tarafdar et al., 2007)*

In what ways, if any, do you feel that GenAI might impact your role or job security in the long term?
How do you personally perceive the potential of GenAI to replace or transform parts of your current job?

---

2. Techno-Uncertainty *(Tarafdar et al., 2007)*

What challenges do you face in keeping up with the rapid development and release of new GenAI tools and models?
How do you usually deal with uncertainties or unclear expectations around the use of GenAI in your work?

---

3. Techno-Overload *(Tarafdar et al., 2007)*

How has the use of GenAI changed your workload or the pace of your daily work?
Have expectations from colleagues or supervisors changed since GenAI became part of your work routine and how do you experience that?

---

4. Techno-Complexity *(Tarafdar et al., 2007)*

Can you describe your experience learning how to use GenAI tools such as ChatGPT effectively in your work?
Which aspects of working with GenAI feel particularly complex or difficult and how do you deal with them?

---

5. Techno-Invasion *(Tarafdar et al., 2007)*

To what extent does GenAI influence your ability to switch off from work or maintain boundaries between work and private life?
Have you noticed that GenAI affects when and how you work, for example outside regular hours or during breaks?

---

6. Techno-Eustress / Positive Stress *(Tarafdar et al., 2019, 2024)*

How do you personally experience working with GenAI, does it motivate, challenge, or excite you in any way?
Can you think of situations where GenAI has helped you grow or accomplish something you’re proud of?

---

7. Compliance, Regulation, Data Security

(based on D’Arcy et al., 2014; Hwang & Cha, 2018; Manduchi et al., 2024; Nah et al., 2023; D’Onofrio, 2024)

How confident do you feel when it comes to compliance and data protection while working with GenAI tools?
What kinds of rules, risks, or uncertainties do you associate with using GenAI in your professional context?

---

8. Unreliability of ICT / GenAI Systems *(based on Ayyagari et al., 2011; Califf et al., 2020; Manduchi et al., 2024)*

What has your experience been regarding the accuracy and trustworthiness of GenAI-generated outputs?
Have you encountered mistakes, hallucinations, or unexpected outputs from GenAI tools and how did you respond?

---

9. Cognitive Impact of GenAI *(based on Caporusso, 2023; Lee et al., 2025)*

To what extent do you feel dependent on GenAI tools in your daily work?
How has your reliance on GenAI changed over time and what does that mean for your own expertise?

In what ways has GenAI influenced your creative thinking or problem-solving at work?

Have there been situations where you felt that GenAI replaced things you used to do yourself and did that affect your skills?

Are there tasks you used to learn from, that are now done by GenAI? What effect does that have on your personal development?
