# Supplementary material for: Technostress and generative AI in the workplace: a qualitative analysis of young professionals
Source: Front Artif Intell. 2025 Dec 12;8:1728881. doi: 10.3389/frai.2025.1728881 (PMC12741148; doi:10.3389/frai.2025.1728881)
Supplement: Supplementary file 2 [file Data_Sheet_2.pdf]

Codebook on the further findings

Category	Subcode	Explanation	Anchor examples
Positive Stress	Work Simplification	Perceptions that GenAI reduces effort, speeds up tasks, or streamlines workflows, making work feel easier or more manageable	*For us, it is more of a benefit than a burden. Generating the same output with less input. It is a useful tool. Our work is becoming increasingly complex. Through ChatGPT we are able to manage this workload." MA12*
Positive Stress	Motivation	Experiences where GenAI use increases enthusiasm or willingness to take on tasks by reducing tedious work or enabling visible performance gains	*"I wouldn't say there is a lot of pressure that I absolutely have to do this to keep up at work. I rather have a certain intrinsic motivation." IT7*
Positive Stress	Enjoyment	Feelings of fun or curiosity when experimenting with GenAI tools, independent of purely instrumental performance outcomes	*I don't see this as a challenge at all but rather have fun with it." IT7*
Positive Stress	Technology Affinity	Generally positive attitude toward digital technologies and GenAI, including interest, openness, and confidence in trying out new tools and features	*"I enjoy testing new things. I'm not overwhelmed, but rather interested." IT7*
Reliability	Control and Verification	Efforts (or need) to check, validate, or correct GenAI outputs as well as perceived responsibility for ensuring final quality	*"I suspect that this ultimately results in a huge maintenance effort because no one on-site properly documents and checks this code if needed. In the worst case, the code does not do what it is supposed to, and no one notices, producing errors" R&D1*
Reliability	Prone To Errors	Perceptions that GenAI frequently produces incorrect, incomplete, or misleading results, requiring additional effort to detect and correct mistakes	*"You always have to be careful about the information you receive, as the texts are not always entirely correct." MA12*

Reliability	Hallucination	Instances where GenAI generates plausible but fabricated information that may be hard to spot	*"Well, when I process an Excel spreadsheet with GenAI, sometimes the data is incorrect or partly fictitious. Of course, you have to look closely." IT5*
Reliability	Bias	Perceived systematic distortions in GenAI outputs that may conflict with users' ethical or professional standards	*"This may not be relevant to everyone, but depending on the model, the manufacturer may have a certain bias." IT8*
Cognitive effects	Dependency	The tendency to rely too much on GenAI for ideas, wording, or decisions. Concerns about "not being able to work without it" or losing autonomy	*"I have definitely gotten worse because I already rely on the AI to take over part of the work for me." R&D3*
Cognitive effects	Skill degradation	Fears that frequent GenAI use reduces one's own skills over time because the tool performs substantial parts of the task	*"if the tools were to suddenly disappear, then I would probably be worried. Then I would have to relearn all that." R&D1*
Cognitive effects	Creativity	The feeling that one's own creativity suffers when many mental tasks are outsourced to GenAI	*"one somehow under-challenges oneself a bit when one or others use artificial intelligence and as a result I believe creativity already shrinks a little bit." MA13*
Cognitive effects	Learning Experiences	The feeling that important learning experiences are being missed because too many important tasks are being solved by GenAI	*"If this technology had not come, I would probably have acquired significantly more skills in programming." R&D1*
Cognitive effects	Critical thinking	Users' reflection on, questioning of GenAI outputs includes concerns that over-reliance may weaken these evaluative capacities	*"Simply not having to think about things again" and that one "also does not have to have all the connections in one's head." MA13*
Compliance	Data Security	Concerns about how input data are handled by GenAI tools, including risks of leakage, misuse, or non-compliance with security policies	*"It already starts with simple things like 'let's write a letter' and then people just copy in all the information." R&D1*

Compliance	Copyright	Worries about intellectual property, authorship, and reuse of GenAI-generated or training data–derived content, especially in professional or commercial contexts	*"We've always freely borrowed ideas, but it used to be a bit easier to determine: does this belong to me or not? Now there's this added uncertainty. We also hand things over to others. When is my idea really mine?" MA14*
Compliance	Regulation	References to external legal frameworks and uncertainty about how these rules apply to everyday GenAI use at work	*"It's really critical when private accounts are used for official purposes. But there are no clear rules. I'd prefer if there were very concrete regulations." R&D3*
Compliance	Trust	The degree of trust users have in GenAI outputs and especially the systems and companies behind them, including perceived reliability, integrity, and alignment with organizational goals	*"When Meta reserves the right to grab the data in order to build its own AI models with it. Against this background, I find it extremely problematic how little is being controlled and regulated." MA14*
Compliance	Ethics	Moral considerations around GenAI use and perceived conflicts with personal or professional values	*"I personally find that in the area of ethics we really have to be careful. The technology is quite new and evolving rapidly. I'm optimistic, but still rather skeptical." FIN10*
Role Shift	Job Change	Expectations or concerns that GenAI will alter job content, required competencies, or employment prospects	*"It will simply change the work, but there will be new tasks, such as sensible prompt design" IT7*
Role Shift	Multi-Level-Responsibility	Perceived ambiguity or diffusion of responsibility for GenAI use and its consequences across different levels	*"Not on a personal level, it's not personal stress, but I think it's more on a company level, it's important that this competence is simply available somewhere in a collective." MA14*
Role Shift	Process problems	Frictions that arise when integrating GenAI into existing workflows and procedures	*"AI tools can sometimes slow down work processes because they make you more aware of their capabilities. For example, you might think, "I could throw that into DeepL or ChatGPT." This, in turn, takes extra time." R&D4*
